# Quercetin and/or Ascorbic Acid Modulatory Effect on Phenobarbital-Induced Sleeping Mice Possibly through GABA_A_ and GABA_B_ Receptor Interaction Pathway

**DOI:** 10.3390/ph14080721

**Published:** 2021-07-26

**Authors:** Rajib Hossain, Khattab Al-Khafaji, Rasel Ahmed Khan, Chandan Sarkar, Md. Shahazul Islam, Dipta Dey, Divya Jain, Farhana Faria, Rukaya Akbor, Olubunmi Atolani, Sónia M. R. Oliveira, Abolghasem Siyadatpanah, Maria de Lourdes Pereira, Muhammad Torequl Islam

**Affiliations:** 1 Department of Pharmacy, Life Science Faculty, Bangabandhu Sheikh Mujibur Rahman Science and Technology University, Gopalganj 8100, Bangladesh; rajibhossainrh021@gmail.com (R.H.); csarkar1053@gmail.com (C.S.); islamshahazulphr04@yahoo.com (M.S.I.); faria.pharma15@gmail.com (F.F.); rukaya.akbor9018@gmail.com (R.A.); 2 Department of Chemistry, Faculty of Arts and Sciences, Gaziantep University, 27310 Gaziantep, Turkey; k.a.alkhafaji@gmail.com; 3 Pharmacy Discipline, Life Science School, Khulna University, Khulna 9280, Bangladesh; raselahmed358@gmail.com; 4 Department of Biochemistry and Molecular Biology, Bangabandhu Sheikh Mujibur Rahman Science and Technology University, Gopalgonj 8100, Bangladesh; diptadey727@gmail.com; 5 Department of Bioscience and Biotechnology, Banasthali Vidyapith, Vanasthali 304022, Rajasthan, India; divyajain31011996@gmail.com; 6 Department of Chemistry, University of Ilorin, Ilorin P.M.B. 1515, Nigeria; atolani.o@unilorin.edu.ng; 7 CICECO-Aveiro Institute of Materials & Department of Medical Sciences, University of Aveiro, 3810-193 Aveiro, Portugal; sonia.oliveira@ua.pt; 8 Hunter Medical Research Institute, New Lambton, NSW 2305, Australia; 9 Ferdows School of Paramedical and Health, Birjand University of Medical Sciences, Birjand 9717853577, Iran; asiyadatpanah@yahoo.com; 10 Department of Medical Sciences, University of Aveiro, 3810-193 Aveiro, Portugal

**Keywords:** ascorbic acid, quercetin, *Mus musculus*, stimulatory-*like* activity, GABA receptor, molecular docking

## Abstract

Depressive disorder is a recurrent illness that affects large numbers of the general population worldwide. In recent years, the goal of depression treatment has moved from symptomatic response to that of full remission. However, treatment-resistant depression is a major challenge in the treatment of depression or depression-related disorders. Consensus opinion, therefore, suggests that effective combined aggressive initial treatment is the most appropriate strategy. This study aimed to evaluate the effects of quercetin (QUR) and/or ascorbic acid (AA) on Phenobarbital-induced sleeping mice. QUR (50 mg/kg) and/or AA (25 mg/kg) with or without intraperitoneally pre-treated with GABA receptor agonist (diazepam: 2 mg/kg, i.p.) or antagonist (Flumazenil: 2.5 mg/kg, i.p.) to underscore the effects, as well as the possible involvement of the GABA receptor in the modulatory action of QUR and AA in sleeping mice. Additionally, an in silico study was undertaken to predict the involvement of GABA receptors in the sleep mechanism. Findings suggest that the pretreatment of QUR and AA modulated the onset and duration of action of the standard drugs in experimental animals. The acute administration of QUR and/or AA significantly (*p* < 0.05) reversed the DZP-mediated onset of action and slightly reversed the duration of sleep time in comparison to the vehicle (control) group. A further combination of QUR or AA with the FLU resulted in an enhancement of the onset of action while reducing the duration of action, suggesting a FLU-like effect on the test animals. In in silico studies, AA and QUR showed good to moderate binding affinities with GABA_A_ and GABA_B_ receptors. Both QUR and AA produced a stimulatory-like effect on mice, possibly through the GABA_A_ and GABA_B_ receptor interaction pathways. Further studies are necessary to verify this activity and clarify the exact mechanism of action(s) involved.

## 1. Introduction

Generally, there are several situations and conditions that stimulate stress to vary degrees in humans [1]. However, anxiety and depression are principal reaction conditions that induce stress and stress disorders or complications [2]. It is estimated that major depressive disorders are expected to be the primary cause of disability in the world by 2030. These disorders are currently considered the most common psychiatric illnesses affecting humans [3]. These are associated with significant disability, socioeconomic burden, and a negative impact on the quality of life [4]. Benzodiazepines (BDZ) and barbiturates are the first-line pharmacological anxiolytics and antidepressants, and more psychoactive medications have been developed in the last few years [5]. Current pharmacological interventions suggest that the drugs used to manage these disorders often have several side effects including drug interactions, delayed response, and even non-response to the treatment [6]. For producing anxiolytic and/or antidepressant drugs, the gamma-aminobutyric acid (GABA) receptor is the most essential target [7]. At the pharmacological and molecular level, the GABA receptor has two subtypes—GABA_A_, and GABA_B_ [8]. GABA_A_ receptors (containing α1, α2, α3, or α5 subunits) are a ligand-gated pentameric architectural ion channel that seems to be sensitive to benzodiazepines and other anxiolytic and hypnotic drugs, whereas GABA_B_ receptors are a type of G protein-coupled heterodimer receptor that has a muscle relaxant site [9]. For example, benzodiazepine receptor agonists exhibiting intense impact at the α1 and/or α5 subunits are more likely to cause drowsiness, ataxia, and amnesia, even though those having increased activity on GABA_A_ receptors possessing the α2 and/or α3 subunits are much more likely to be calming [10].

Cognitive impairment and depression are prominent symptoms of psychiatric systemic lupus erythematosus, with cognitive impairment being the most prevalent symptom [11,12,13,14,15]. In lupus erythematosus, the involved in the pathogenesis pathways of cognitive deterioration and depression are yet unknown. In depressive disorder, cognitive dysfunction might have been a mediating factor of functional disability. Attention, verbal and nonverbal learning, short-term and working memory, visual and auditory processing, problem-solving, processing speed, and motor function are all examples of cognitive dysfunction [16,17]. Frampton (2016) revealed that cognitive dysfunction is exhibited in depressive conditions [17], which may attenuate learning memory, concentration, and finally dementia [18]. Furthermore, several studies have suggested that depression increases the course of dementia in people with moderate cognitive impairment [19]. The combined or individual causes of co-occurring depressive and cognitive dysfunction are poorly understood [20].

Additionally, in depression, cognitive biases and cognitive deficits are the two vital classes of cognitive dysfunction. One (cognitive biases) includes erroneous processing of information or a bias towards negative stimuli, whereas the other (cognitive deficits) reduces concentration, short-term memory, and executive functioning of the brain [21,22,23,24,25,26]. When patients employed either high levels of avoidance or low levels of active coping, cognitive impairment was most likely to be linked to depression [27]. Some conventional antidepressant medications (SSRIs and SNRIs) may improve cognitive deficits associated with depression [17]. Significantly, cognitive dysfunction (also known as psychosocial functioning) can remain even after patients have satisfied traditional psychological distress remit requirements [24,28,29].

Phenobarbital (barbiturate) is a non-selective central nervous system (CNS) depressant, primarily used as a sedative-hypnotic, and also as an anticonvulsant in sub-hypnotic doses. Despite the clinical efficacy, most drugs in this class have been seen to exert several side effects, including sedation, muscle relaxation, anterograde amnesia, and an increased risk of accidents [30,31]. Chronic use of these drugs can lead to psychomotor effects, paradoxical reactions, tolerance, teratologic risk, and dependence [32]. Currently, the goal of depression treatment has moved from mere symptomatic response to that of full remission (i.e., minimal/no residual symptoms). Unfortunately, treatment-resistant depression is a major obstacle to the predominant treatment strategies for depressive disorders. On the other hand, the longer it takes to get into remission, the greater the risk of growing treatment resistance to a particular strategy. Thus, the combined aggressive initial treatment should be an appropriate strategy in this case [33].

Natural products are used as the primary source of medical intervention worldwide; with up to 38–80% (depending on the country of origin) of the population directly or indirectly utilizing photo-derived therapeutic agents to address various healthcare needs [34]. Thus, natural products made up of various constituents, such as flavonoids, glycosides, alkaloids, terpenoids, among others, are one of the promising and alternative routes in the drug discovery and development context, accounting for a vast quantity of the approved chemotherapeutic agents being used in the management of anxiety and depression-like illnesses [35]. Although it remains uncertain whether the human lifespan could be prolonged by increased flavonoid intake [36], cumulative studies suggest that plant-derived flavonoids have diverse medicinal properties that can be used for the treatment of several diseases and disorders, including microbial infection, inflammation, pro-oxidant-related diseases, malaria, mutagenic toxicity, infertility [37,38,39], cancer, neurodegenerative or cardiovascular diseases [40].

**Figure 1:**
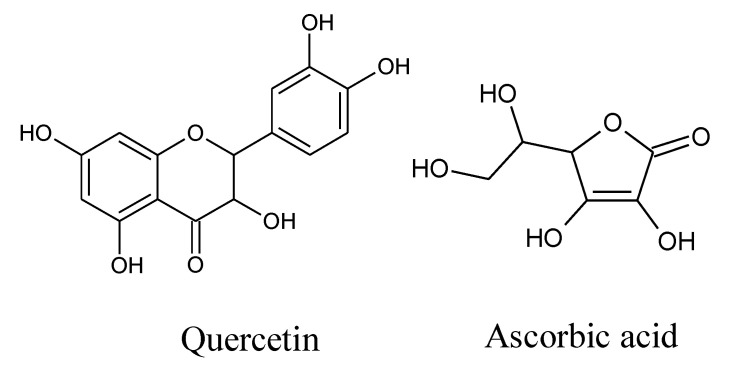
Chemical structure of quercetin and ascorbic acid.

Quercetin (QUR, chemically named 3, 3′, 4′, 5, 7-pentahydroxyflavone) [41,42], is a bitter plant-derived polyphenol (flavonol). Quercetin (Figure 1) is found in many fruits, vegetables, leaves, grains, red onions, and kale, among others. It is estimated that the average person consumes 10–100 mg of QUR from their daily diet [43]. QUR has many important biological activities [44], including antioxidant [45], anti-inflammatory [43], neuroprotective [46,47], anticancer properties [48], anxiolytic-*like* effects [49], and so on. In a recent study, quercetin 4’-O-glucoside (quercetin derivatives) has been found to exert an anti-depressant-like effect on mice, possibly via prevention of brain oxidative stress and restoration of serotonin levels by inhibiting MAO-A activity [50]. In another study, QUR supplementation with levetiracetam (a medication used to treat epilepsy) has been seen to ameliorate depression associated with epilepsy in a dose-dependent manner in male Swiss mice [51]. Some studies suggested that QUR could interact with the GABA_A_ α5 receptor to reduce seizures [52], while with the GABA_A_ receptor β1 and β3 subunits for its anti-epileptic effect in experimental animals [53].

Ascorbic acid (AA, also called vitamin C, Figure 1) is a frequently used vitamin by human beings [54]. It was initially identified as an agent to prevent scurvy disease, and now it is well known for its strong antioxidant properties [55,56]. Recently, AA has been identified as an important vitamin that can be used to manage all kinds of stressful conditions that are linked to inflammatory processes and immunity. Therefore, it can be considered as a combination component with other substances like antioxidants and drugs [57]. Scientific reports suggest that AA has some other beneficial effects on the management of psychiatric disorders [58], including depression [59,60], anxiety and schizophrenia, and neurodegenerative diseases (Alzheimer’s disease, Parkinson’s disease, Huntington’s disease, multiple sclerosis, and amyotrophic sclerosis) [61]. In a recent study, AA was found to alter lead-induced reduction of Purkinje cells and reduced expression of the synaptic marker (synaptophysin), gamma-aminobutyric acid (GABA)-synthesizing enzyme (glutamic acid decarboxylase 67), and axonal myelin basic protein in rat brain [62].

This study aimed to evaluate the effects of QUR and/or AA on Phenobarbital-induced sleeping *Swiss* mice. Additionally, our goal is to understand the possible involvement of GABA receptors in the modulatory action of QUR and AA (independent and combined) with the GABA agonist or antagonist drug-receptor through molecular docking studies.

## 2. Results

### 2.1. Animal Study

In the Phenobarbital sleeping test, the administration of DZP (2 mg/kg) reduced the onset of sleep (latency), while augmenting the sleeping time compared to the control (vehicle) group. On the other hand, the test drugs, QUR, and AA increased both the onset and duration of sleep time in comparison to the NC group. However, pretreatment of QUR or AA in the combined groups caused a significant (*p* < 0.05) reduction in latency periods, but a slight increase in sleeping time. AA alone or in its combination (DZP + AA) exhibited a reduced onset of action and decreased duration of action in experimental animals than the QUR-treated groups (Gr.-III and Gr.-V). A pretreatment of AA-plus QUR was found to reduce the latency period significantly (*p* < 0.05) in the DZP group when compared to the QUR and AA groups. However, the duration of sleep in this group was significant only when compared to the NC and QUR groups (Table 1).

**Figure 2:**
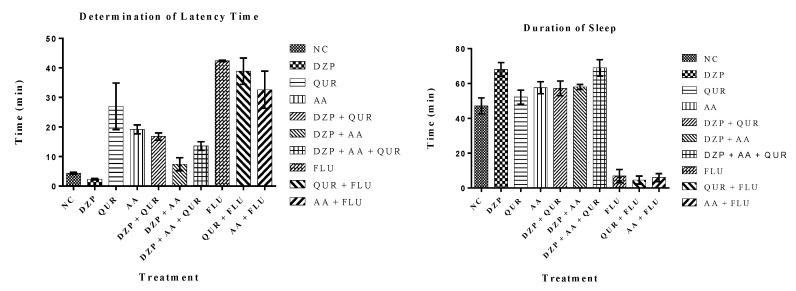
Determination of latency and duration of sleep on the phenobarbital-induced sleeping mice.

Table 1 also suggests that animals pretreated with the FLU (2.5 mg/kg) took the maximum time to sleep (42.38 ± 0.23 min) and showed the lowest duration of sleep (06.99 ± 3.67 min) (Figure 2). Pretreatment of QUR or AA with the FLU was found to increase the QUR- or AA-mediated latent period, while a decrease in sleeping time in experimental animals compared with the positive control.

### 2.2. In Silico Study

#### 2.2.1. GABA Homology Model

**Figure 3:**
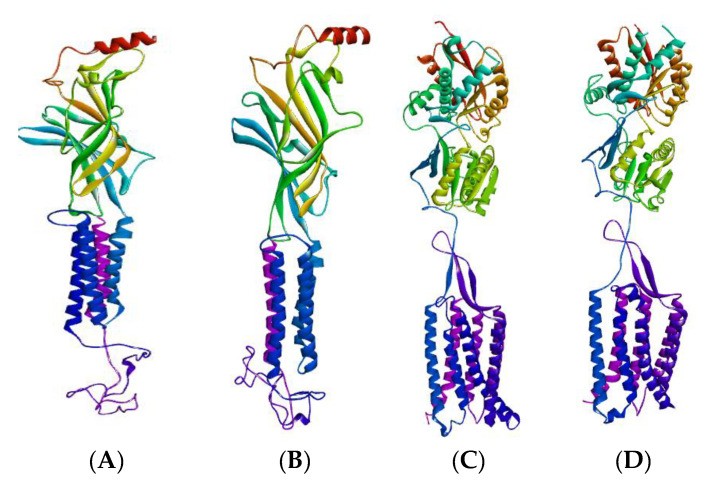
Homology model of GABA receptors (**A**) GABA-A2, (**B**) GABA-A5, (**C**) GABA-B1, and (**D**) GABA-B2 through the Swiss model.

**Figure 4:**
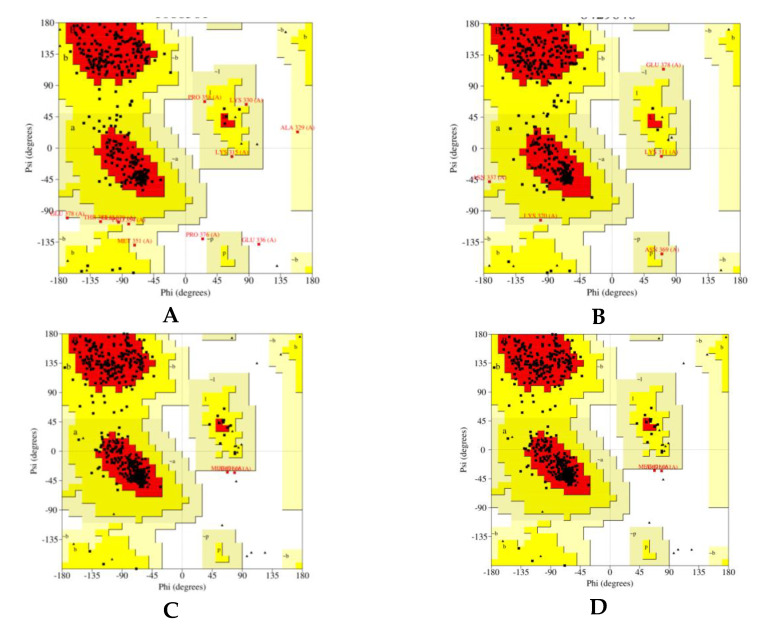
The optimized model of human GABA receptors (**A**) A2, (**B**) A5, (**C**) B1, and (**D**) B2 using PROCHECK.

Homology modeling has developed into an effective structural biology tool, greatly shrinking the distance between experimentally described protein structures and recognized protein sequences [63]. Using completely automated frameworks and databases, the homology modeling process is optimized and standardized, enabling even those without a specialized computational background to create accurate protein maps and have a fast and clear reference to modeling findings, representation, and evaluation [64,65]. The amino acid sequences of GABA (A2, A5, B1 and B2) collected by Uniprot (Uniprot accession ID: P47869, P31644, P18505, P47870, respectively), were subjected to the NCBI Blast Program for selection of the closest homologous template homology model of GABA (A2, A5, B1, and B2) and were generated by the Swiss model. Figure 3 shows the 3D homology model of the GABA receptors. Optimization of the GABA models was achieved by using the Swiss-PDB Viewer software package (version 4.1.0) before docking, whereas validation of these GABA homology models was acquired through the use of the Ramachandran plot performed by PROCHECK [66] and illustrated in Figure 4.

The Ramachandran plot is a simple way to see how a protein structure’s torsion angles are distributed. It also gives an overview of the allowed and disallowed regions of torsion angle values, which is useful when evaluating the quality of protein three-dimensional structures. The phi-psi torsion angles for all residues in the structure are seen in the Ramachandran plot (except those at the chain termini). Triangles are used to represent glycine residues because they are not confined to the plot sections designated for one of the other side chain variants. The plot’s coloring/shading depicts the several locations characterized: The darkest portions (shown in red) correlate to the “core” sections, which reflect the most advantageous phi-psi value configurations. In an ideal world, over 90% of the residues would have been found in these “core” areas. One of the best indicators of stereochemical integrity is the proportion of residues in the “core” areas (Figure 4).

From Ramachandran plot statistics, it has been found that residues in the most favored regions are about 92.0%, 90.8%, 91.2%, and 90.5% for GABA A2, A5, B1, and B2, respectively.

#### 2.2.2. Interaction of Quercetin (QUR) with GABA Receptor

**Figure 5:**
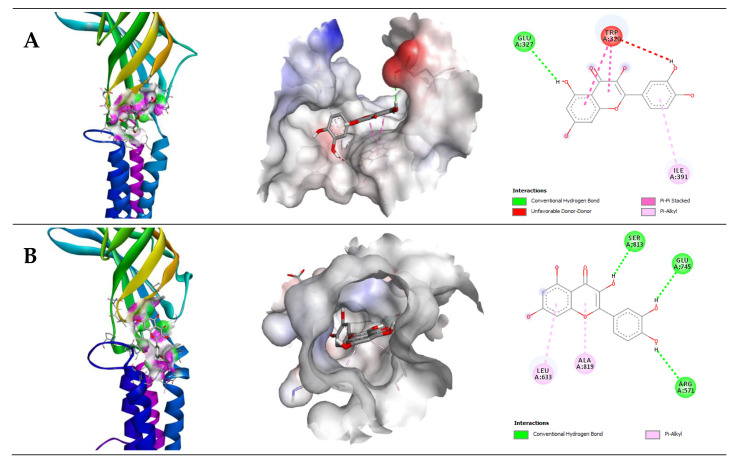

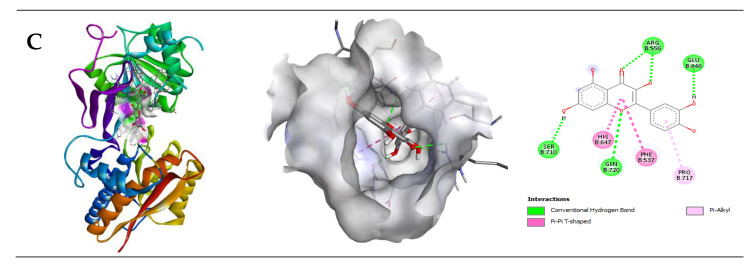
Molecular docking interaction of (**A**) GABA(A5), (**B**) GABA(B1), and (**C**) GABA(B2) receptor with quercetin (QUR).

Quercetin displayed good binding affinities with GABA receptor subunits such as A5, B1, and B2. The binding affinities were −6.9, −8.4, and −8.2 kcal/mol, respectively. Quercetin is bound to the GABA (A5) subunit through one H-bond with Glu327, one pi-pi, and one pi-alkyl bond with Ile391, Trp320, respectively. Furthermore, QUR displayed a binding affinity with GABA (B1) and GABA (B2) through three H-bonds with Arg571, Glu745, Ser813; two pi-alkyl bonds with Ala819, Leu633, and four H-bonds with Arg556, Gln770, Glu646, Ser710; two pi-pi bonds with His647, Phe537; and one pi-alkyl with Pro717, respectively (Table 2). The 2D and 3D structures of non-bond interactions of QUR with GABA receptor subunits are shown in Figure 5.

#### 2.2.3. Interaction of Ascorbic Acid (AA) with GABA Receptor

**Figure 6:**
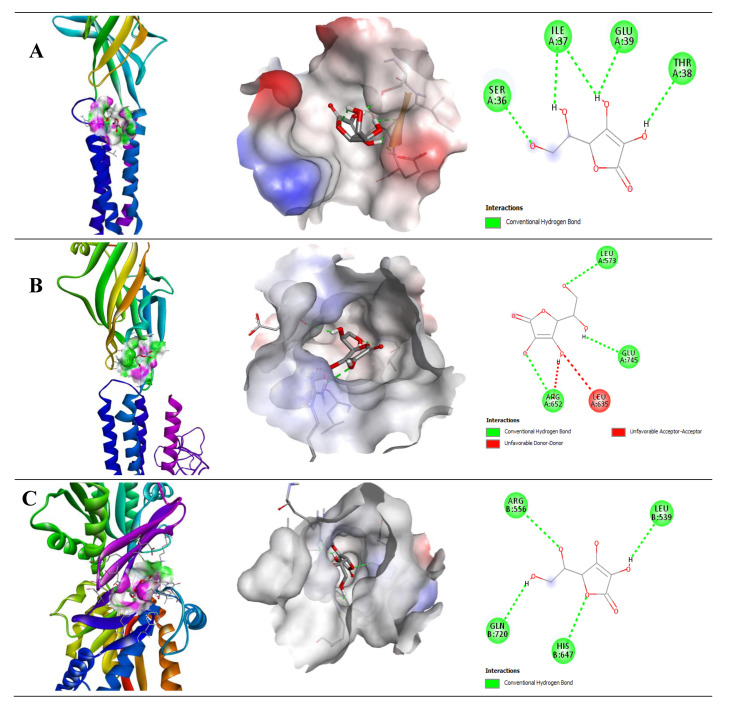
Molecular docking interaction of (**A**) GABA-A2, (**B**) GABA-B1, and (**C**) GABA-B2 receptor with ascorbic acid.

The binding affinities of ascorbic acid (AA) were −5.0, −5.5, and −5.3 kcal/mol with GABA A2, B1, and B2, respectively (Table 3). In comparison with QUR, AA showed moderate binding affinities with GABA receptor subunits, including GABA A2, B1, and B2, respectively. Only AA showed other than H-bond with Leu635 amino acid residue in the GABA (B1) receptor, but it also showed a binding affinity with the GABA (B1) receptor through H-bond with Arg652, Glu745, and Leu573. Moreover, AA generated four H-bonds with Ile37, Glu39, Ser36, and Thr38 in GABA (B1), and Arg556, Gln720, His647, and Leu539 in the GABA (B2) receptor subunit. The 2D and 3D structures of non-bond interactions of QUR with GABA receptor subunits are shown in Figure 6.

#### 2.2.4. MD Simulation Study

**Figure 7:**
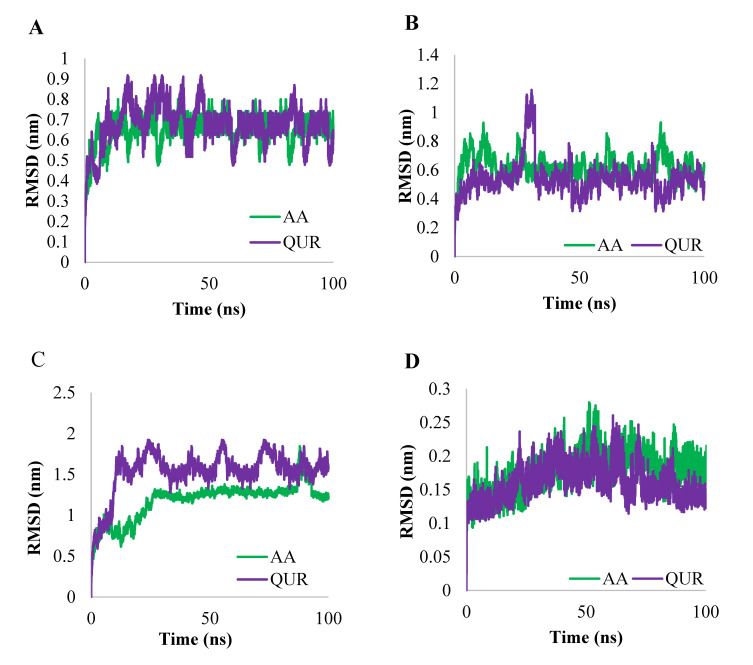
Time evolution of the RMSD values for the complexes of (**A**) backbone atoms of GABA (A2) in AA-bound and QUR-bound forms, (**B**) backbone atoms of GABA (A5) in AA-bound and QUR-bound forms, (**C**) backbone atoms of GABA (B1) in AA-bound and QUR-bound form and (**D**) backbone atoms of GABA (B2) in AA-bound and QUR-bound form.

The RMSD values were calculated for AA-bound and QUR-bound GABA (A2), GABA (A5), GABA (B1), and GABA (B2) proteins and are shown in Figure 7. The first protein of selected targets is GABA (A2), where the RMSD values for the protein’s backbone atoms of MD trajectories for GABA (A2) in AA-bound form and QUR-bound form are shown in Figure 7A. The AA-bound GABA (A2) and QUR-bound GABA (A2) complexes reached equilibrium after passing 15 ns and the two systems showed stable fluctuation (0.5 nm to 0.9 nm). The target GABA (A5) exhibited a different fashion of dynamics, where the RMSD of backbone atoms of QUR-bound GABA (A5) and AA-bound GABA (A5) reached equilibrium after 10 ns. Overall, the RMSD of backbone atoms of QUR-bound GABA (A5) fluctuated less than the RMSD of backbone atoms of AA-bound GABA (A5) (Figure 7B). Inversely, the RMSD of the backbone of AA-bound GABA (B1) fluctuated less than QUR-bound GABA (B1) (Figure 7C). Also, the RMSD of backbone atoms of AA-bound GABA (B2) and QUR-bound GABA (B2) has the same style of dynamics (Figure 7D).

**Figure 8:**
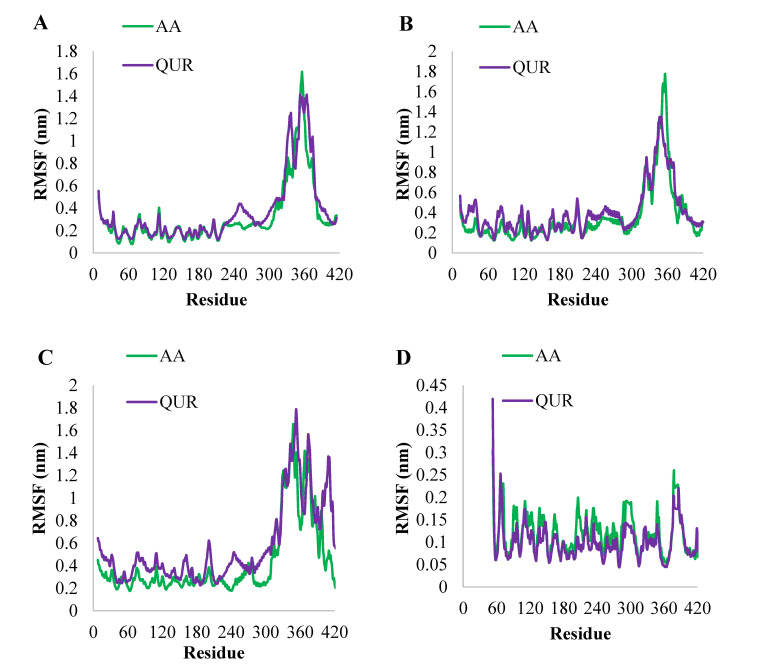
Comparative RMSF value of the complex of (**A**) backbone atoms of GABA (A2) in AA-bound and QUR-bound forms, (**B**) backbone atoms of GABA (A5) in AA-bound and QUR-bound forms, (**C**) backbone atoms of GABA (B1) in AA-bound and QUR-bound form, and (**D**) backbone atoms of GABA (B2) in AA-bound and QUR-bound form.

To check the influence of structural flexibility AA or QUR on the rest of the proteins GABA (A2), GABA (A5), GABA (B1), and GABA (B2), the average RMSF of the entire complex backbone is calculated during 100 ns and presented in Figure 8. The residue-wise fluctuation of AA-bound GABA (A2) and QUR-bound GABA (A2) were plotted and presented in Figure 8A. The compound, AA, reduced significantly the RMSF for the backbone of GABA (A2) 230–279 amino acids, and this reduction in RMSF was due to the binding of AA to the binding site, except for the nature of residual fluctuations of AA-bound GABA (A2) very near to residual fluctuations of QUR-bound GABA (A2) backbone atoms (Figure 8B). But it is worthwhile to note that the AA reduced the overall residual fluctuations of GABA (A5) and GABA (B1) when compared with the QUR effect upon the dynamics of GABA (A5) and GABA (B1) (Figure 8B,C). However, QUR was able to reduce the residual fluctuations of GABA (B2) more than AA (Figure 8D).

#### 2.2.5. Binding Free Energy (MM-PBSA) Analysis

In order to understand the biophysical basis for molecular recognition of AA and QUR with targets (GABA (A2), GABA (A5), GABA (B1), and GABA (B2)), the MM-PBSA program provides various individual components, such as ∆G_vdW_, ∆G_elec_, ∆G_pol,_ and ∆G_nonpol_, which are used to calculate the total binding free energy (∆E MMPBSA). It can be concluded from Table 3 that the intermolecular van der Waals interaction (∆G_vdW_), electrostatic interaction (∆G_elec_), and solvation energy are non-polar (∆G_nonpol_), and the promotion of free polar solvation energy (∆G_pol_) is unfavorable.

Further, Table 4 reveals that the estimated binding free energy (∆E MM-PBSA) of GABA A2-QUR is the highest (−33.538 kJ/mol) despite unfavorable contributions from ∆G_pol_ (53.211 kJ/mol). In the case of the GABA B1-QUR complex, the polar contribution has the highest positive value) ∆G_pol_ = 142.703 kJ/mol. QUR interacted more strongly than AA when it interacted with GABA (A2) ((∆E MM-PBSA = −33.538 kJ/mol), GABA (B1) (∆E MM-PBSA = −25.083 kJ/mol), and GABA (B2) ((∆E MM-PBSA = −16.850 kJ/mol). While AA interacted more strongly than QUR with GABA (A5) (∆E MM-PBSA = −32.068 kJ/mol), generally, the binding affinities of AA and QUR with targeted proteins are close to each other.

## 3. Discussion

Sedative and anxiolytic drugs, such as BDZ, exert their action through GABA upon the GABA_A_ receptor [67]. The Phenobarbital-induced sleep test evaluated the possible anti-sedative effects of QUR and AA. In this test, CNS depressants and sedative drugs classically decrease sleep latency and increase sleep time.

In the human body, AA may act as an endogenous ligand that can potentiate GABA neurotransmission in the CNS [68]. In the intra-ventral segmental area, GABA (A) receptors play a vital role in the modulation of narcotic drugs in the nucleus accumbens of freely moving rats [69,70]. AA at 0.5 mM concentration exhibits a neuroprotective effect through interaction with the GABA (B) receptor [71]. In rat brain cortical synaptosome, at 10^–3^ M concentration, AA strongly abrogates, while at 10^–6^ M concentration, it stimulates 3H-GABA binding capacity [72]. Recent findings by Rosa indicated that AA exerts an anti-depressant effect on mice at a concentration of 1 mg/kg, p.o., possibly by interacting with GABA (A), and (B) receptors [73]. On the other hand, AA at 100 mg/kg, oral administration, has been found to abrogate the ethanol-induced increased expression of the GABA (B1) receptor [74]. Furthermore, it has been shown that AA exerts an anti-convulsion activity at a dose of 250 mg/kg, i.p. administered in rats via activation of the GABAB1R/CaMKII/CREB pathway, which suggests that AA has therapeutic potential in epilepsy [75]. A recent study showed that QUR possesses anti-seizure effects, possibly by interacting with the GABA_A_-α5 receptor [76].

Cognitive impairment is induced in brain cells via oxidative stress. Several studies have reported that QUR reverses cognitive dysfunction by protecting the PNS and CNS neurons in the brain [77,78]. QUR has strong antioxidant properties. Therefore, QUR may suppress oxidative stress by blocking several oxidative enzymes and inducing antioxidant enzymes [78]. It has been observed that QUR protects neurons by blocking oxidative stress, neuroinflammation, and memory deficits in a dose-dependent manner [79]. QUR could enhance memory retrieval [80]. Furthermore, quercetin drastically enhances the locomotor activity of mice in open field tests [81]. Besides, AA also possesses good antioxidant properties [82] with increasing SOD and GPx effects on the brain [83]. Moreover, it can modulate glutamatergic, cholinergic, dopaminergic, and GABAergic neurotransmission processes, and help neurons differentiate, mature, and survive. Further, AA blocks inflammation in the astrocytes and prevents neuronal cell damage [58]. Another study reported that AA protects neuron cells from damage in the brain hippocampus and reverses memory deficits by regulating oxidative stress [84]. Delrobaei et al. (2019) revealed that AA attenuates estradiol for the amelioration of cognitive impairment. It also improves learning memory (300 and 500 mg/kg) and working memory (100 and 500 mg/kg) [83].

Some studies have demonstrated that AA possesses an antidepressant-like effect through involving 5-HT1A, 5-HT2A/2C, and 5-HT3 serotonin receptor activation. In the tail suspension test, AA provides a synergistic effect with some conventional antidepressant medications (fluoxetine, imipramine, and bupropion) [85,86]. Thus, it should be possible for QUR to improve the cognitive impairment in depressive disorder when co-administered along with ascorbic acid in the mouse model.

Due to the wide range of neurotransmission activity mediated by GABA neurons, it is becoming evident that a GABAergic pathway imbalance, and therefore an E:I misproportion, can be involved in the pathogenesis of a variety of mental illnesses, particularly depression [87]. Humans may develop depression as a result of changes in the brain caused by stress. Scientists have been studying the impact of prolonged stress on cognitive function and neuroplasticity in rats as a model for human brain research [88].

Via modulation of the hypothalamus-pituitary-adrenal (HPA) axis, GABAergic neurons play a significant role throughout the termination of the stress reaction, and disturbance of this legislative reaction relates to the aberrant consequences of psychological distress treatment. In the brain, chronic stress promotes the transmembrane K-Cl cotransporter (KCC2) to be downregulated, leaving GABA inputs inefficient for HPA axis neuronal suppression [89]. Furthermore, elimination or modification of the GABA_A_ γ 2 receptor subtype (heterozygous γ2 knockout: γ2+/−) diminishes GABA_A_ receptor interaction and, as a consequence, HPA axis stimulation, contributing to anxiolytic and pro-depressive responses [90,91,92,93].

As a result, genetic changes in GABA_A_ receptor subtypes are increasingly being utilized to investigate the role of the GABAergic pathway in the pathogenesis of depression and anxiety, as well as possible treatment methods. Additionally, chronic stress also reduced GABAergic axon connectivity and efficacy, as well as GAD67, VGAT, and GAT3 expression [94,95]. Stress produces substantial changes in the GABAergic cascade, which can lead to aberrant cognitive and synapse reflexes, such as procedures performed on dendritic rearrangement [94], as well as changes in electrophysiological posts [96,97]. These findings back up the theory that stress produces significant systemic changes.

According to Godfrey and his colleagues, GABAergic receptor malfunction is connected to the development of depression, and normalizing GABA is connected to the remission of depressive signs and symptoms [98]. GABA levels have been found to be lower in depressed people in investigations [99].

Thus, our study agrees with the QUR-mediated calming effects in Swiss mice, possibly through the GABA_A_ receptor interaction pathway. GABA_A_ receptor subtype α2 mediated anxiolytic actions, whereas α3 and α5 provide myorelaxant actions [67]. DZP is a medicine of the benzodiazepine family, acting on the GABA_A_ receptor and producing a calming effect [100]. The findings suggest that animals in the QUR and AA groups increased the onset and duration of sleep time as compared to the NC group animals, suggesting a possible stimulatory effect. However, the effects of QUR and AA were not stronger than the standard GABA receptor agonist DZP group. Interestingly, QUR and/or AA, when co-treated with DZP, attenuated the sedative effect of DZP. In this study, QUR plus AA co-treated with DZP was found to increase the latency period that was seen in the DZP group and increased the duration of sleep compared to Gr-I to Gr.-VI, suggesting a modulatory effect of the DZP-mediated calming effects on the animals. On the other hand, FLU is a selective GABA_A_ antagonist [101], essentially used as an antidote in the treatment of benzodiazepine overdoses [102]. FLU reverses the effects of benzodiazepines by competitive inhibition at the GABA_A_ binding site of benzodiazepine receptors that possess the ability to induce anxiety [103]. Moreover, FLU also binds to GABA_A_ to induce its anxiolytic effects [104].

In this study, FLU was seen to augment antagonizing the effects of Phenobarbital-induced sleeping activity in both the QUR and AA groups. Furthermore, several studies suggested that GABA (A1), (A2), (A5), (B1), and (B2) exhibited anti-depressive activity in humans [7,105]. A molecular docking study was performed on the GABA receptor. In Silico studies suggested that both AA and QUR interact with GABA (A1), GABA (A5), GABA (B1), and GABA (B2) receptor subunits. In this study, it was revealed that AA and QUR showed an antagonistic effect on mice models.

The molecular dynamics model (MD) is one of the most widely used calculation methods in the study of biological systems. It is also one of the valuable methods for understanding the dynamic behavior and stability of interactions. From rapid internal movement to slow conformational changes, it is complicated at different times [106]. Our analysis of MD results shows that (Figure 7 and Figure 8) AA and QUR bind firmly to the active sites of GABA (A2), GABA (A5), GABA (B1), and GABA (B2) proteins. RMSD and RMSF also showed that both AA and QUR have nearly similar effects on the dynamic behavior of targeted proteins. Furthermore, MM-PBSA calculations were used to analyze the binding stability of AA and QUR with targeted (GABA) proteins. As illustrated in Table 4, reveals that AA and QUR have similar binding affinities. The general analysis of in silico results has been confirmed, and the in vivo results (animal studies) are consistent with the in silico data, and it is clearly shown that QUR and/or AA can be used as Phenobarbital-induced sleeping mice.

Therefore, this study demonstrated that when QUR and/or AA are co-administered, they may interact with GABA_A_ and GABA_B_ activation and produce a modulatory effect in the mouse experimental model.

## 4. Materials and Methods

### 4.1. Animal Model Study

#### 4.1.1. Chemicals and Reagents

Quercetin (QUR) and ascorbic acid (AA) were purchased from Merck (Mumbai, India). The sources of phenobarbital (Barbit) and diazepam (DZP) (Sedil) were Incepta Pharmaceuticals Ltd. (Dhaka, Bangladesh) and Square Pharmaceutical Ltd. (Pabna, Bangladesh), respectively, while flumazenil (FLU) (Anexate) was purchased from Roache Pharmaceuticals Ltd., Basel, Switzerland.

#### 4.1.2. Experimental Animals

Adult Swiss albino mice (26–30 gm) of either sex have been used in the experiment, and they have been acquired from Jahangirnagar University’s (JU) livestock supply division in Savar, Dhaka. Animals were housed in sterilized polypropylene cages with sterilized husk matting in a comfortable environment (temperature: 25 ± 2 °C, humidity: 50 ± 5%, and 12 h light/dark cycles). Animals were freely accessed to standard pellets as a basal diet and water *adlibitum*. All animals were acclimatized for 2 weeks before starting the study. They were deprived of food before 12 h of the test. Then, animals were randomized and divided into experimental and control groups, each containing 5 animals. The study methodology was approved by the University Research Ethical Committee (UREC) for Animal Subject Research at the Bangabandhu Sheikh Mujibur Rahman Science and Technology University (BSMRTU), Gopalganj-8100, Bangladesh (Approval No. 201511009021; Approval date: 1 March 2019) operating according to the CIOMS and ICLAS international guiding principles for biomedical research involving animals of 2012.

#### 4.1.3. Phenobarbital-Induced Sleeping Test

Phenobarbital sodium was the test compound in the evaluation of Phenobarbital-induced sleeping time in animals. The first four groups (Gr.-I to Gr.-IV) were treated with distilled water (10 mL/kg), diazepam (DZP, 2 mg/kg), quercetin (QUR, 50 mg/kg), and ascorbic acid (AA, 25 mg/kg), respectively. Animals (mice) were given phenobarbital sodium (30 mg/kg, i.p.) after thirty (30) minutes of administration, and the sleep latency was calculated as the time between both the injection of Phenobarbital and the disappearance of the righting reflex. The period seen between the absence of such a restoring response and its voluntary recuperation was documented as sleeping time [107].

To evaluate the combined effects of QUR and/or AA with DZP, three groups (Gr. V to Gr. VII) were treated with DZP + QUR, DZP + AA, and DZP + QUR + AA, respectively. In this case, QUR and/or AA were administered 15 min before the DZP administration. Thirty minutes after the DZP administration, the animals received a Phenobarbital injection. To evaluate if QUR and AA effects were mediated by GABA/benzodiazepine receptors, three groups (Gr. VIII to Gr. X) of mice were pretreated with flumazenil (FLU, 2.5 mg/kg, i.p.). Thirty minutes after the treatment, the latency and sleeping time of animals were noted to be similar to a previously performed protocol. All treatments were given via the intraperitoneal route. Groups receiving treatments are shown in Table 5.

#### 4.1.4. Statistical Analysis

All values are expressed as the mean ± standard error of the mean (SEM). The data were analyzed utilizing analysis of variance (ANOVA) followed by *t*-Student–Newman–Keuls’s as a posthoc test by using GraphPad Prism software (version: 6.0) considering *p* < 0.05 at a 95% confidence interval.

### 4.2. Molecular Docking (In Silico) Study

#### 4.2.1. GABA Homology Model and Macromolecule

Homology modeling of human γ-aminobutyric acid (GABA) was performed by the Swiss model [108]. Before modeling, the sequence was collected from UniProt [109] followed by BLAST analysis using the NCBI BLAST [110] program to choose the template. PROCHECK [111] was employed for the validation of the Homology Model. Molecular docking of AA and QUR compounds was performed to shed light on the binding mode of GABA.

#### 4.2.2. Ligand Preparation

For energy minimization of the crystal structure, we utilized the Swiss-PDB Viewer software package (version 4.1.0) before docking. Furthermore, the chemical structure of ascorbic acid (AA) (PubChem ID: 54670067), and quercetin (QUR) (PubChem ID: 5280343), (Figure 1) was obtained from the PubChem repository sample in the ‘sdf’ file format. All internal energies of the ligands were optimized by using Chem3D Pro12.0 program packages [112].

#### 4.2.3. Docking Protocol

In drug discovery, computational molecular docking simulation is a computerized approach towards drug design. This approach is being used to estimate the pharmacodynamic characteristics of an active substance (ligand) by evaluating and positioning compounds to target binding sites [113] by the PyRx-virtual screening tool. Docking results determine the measure of ligand interaction with the active site of the targeted protein. The active sites are the coordinates of the ligand in the original target protein grids [114] through PyMol and Drug Discovery Studio version 4.5 is used for scrutinizing these active binding sites of the target protein [115].

#### 4.2.4. Molecular Dynamic (MD) Simulation Study

Molecular dynamics (MD) simulation is a basic tool to clarify the binding affinity of small molecules at the binding site and to study the stability of the complex [116]. It gives a picture of the conformational changes or behavior with a time axis, which is used to determine whether the target ligand complex is stable. In the current work, MD simulations of the selected protein-AA or protein-QUR complexes were performed. The GROMACS 2020.1 software package [117] to run MD simulations was used. The Charmm 27 force field [118] was used to parameterize the 3D structure of proteins, while the Swiss Param web-server [119] was used to generate the parameters and topology of AA and QUR. Choose a solvent for the three-point transfer of intermolecular potential (TIP3P) [120] to dissolve the protein-AA or QUR complex. If necessary, the protein-AA or QUR system can be neutralized by adding sodium and chloride ions. The next step is to use the steepest descent algorithm to minimize the energy of protein-drug systems to a tolerance value of 1000 kJ/mol·nm. Then, to use the equilibration with the role of position restraint on the protein molecules for 0.1 ns, the use of NVT and NPT ensembles. Particle Mesh Ewald (PME) was employed to evaluate all of the electrostatic interactions of biological systems. The next step is to perform MD simulations without being restricted by protein or amygdaline molecules. Finally, MD simulations were performed on a time scale of 100 ns (3 fs time step). According to the MD simulation results, we calculated various features using a specific scheme, including the root-mean-square deviation (RMSD) using gmx RMS and the root-mean-square fluctuation of residues (RMSD) using gmx RMSF.

#### 4.2.5. Molecular Mechanics/Poisson-Boltzmann Surface Area (MM-PBSA) Calculations

In addition to molecular dynamics, molecular mechanics/Poisson-Boltzmann surface area was used to determine the thermodynamic stability of QUR or AA at the target binding site, and to test the overall binding affinities between QUR or AA with chosen targets. The calculations were obtained using the g_mmpbsa [120] script tool. This method is the average of two energy values. The first is the solvation energy, and the second is the potential energy in a vacuum.

∆E (MM-PBSA) = ∆E_MM_ + ∆G_solvation_ (1)

∆E_MM_ and ∆G_solvation_ are the vacuum potential energy and free solvation energy in Equation (1), respectively. The molecular mechanical energy (∆E_MM_) is obtained from the electrostatic component (∆E_ele_) and the van der Waals interaction (∆E_vdW_). The solvation energy is calculated from the polar solvation energy ∆G_pol_ and the non-polar energy ∆G_nonpol_. ∆G_pol_ is calculated using the Poisson–Boltzmann equation (PB) and ∆G_nonpol_ is calculated based on the solvent-accessible surface (SASA), and MM-PBSA calculations were done for the last 20 ns of MD trajectories.

## 5. Conclusions and Final Considerations

In the Swiss mouse model, both QUR and AA altered the start and duration of the effect of the conventional calming (DZP) and anti-calming (FLU) drugs. Their effects, however, were less than those of conventional medicine. In Phenobarbital-induced sleeping mice, the effects of FLU were shown to modify the pretreatment of QUR or AA. Importantly, our data indicate that acute QUR and/or AA administration has a depressant-like impact on mice. The data further back up the theory that QUR and AA interact with the GABAA and GABAB receptors to generate stimulatory-like actions, most likely at receptor subtypes like GABA (A1), GABA (A5), GABA (B1), and GABA (B2). However, more research with sub-chronic and chronic treatments is required to confirm this performance and pinpoint the specific mechanism (s) of action. In this approach, our findings might be applied in clinical practice.

## Figures and Tables

**Table 1:** Effects of quercetin and/or ascorbic acid and the controls on the phenobarbital-induced sleeping mice.

Treatment Groups	Latency (min)	Duration of Sleep (min)
NC	4.35 ± 0.32	47.13 ± 4.56
DZP	2.36 ± 0.21 ^acd^	68.03 ± 3.91 ^acd^
QUR	27.00 ± 7.91	52.20 ± 4.02 ^a^
AA	19.20 ± 1.52 ^c^	57.60 ± 3.40 ^ac^
DZP + QUR	16.80 ± 1.24 ^cd^	57.20 ± 4.26 ^ac^
DZP + AA	7.40 ± 2.22 ^cd^	58.00 ± 1.46 ^a^
DZP + AA + QUR	13.60 ± 1.44 ^cd^	69.00 ± 4.71 ^acd^
FLU	42.38 ± 0.23	06.99 ± 3.67
QUR + FLU	38.89 ± 4.45 ^cd^	04.58 ± 2.29
AA + FLU	32.61 ± 6.30	06.02 ± 2.31

Values are mean ± SEM (*n* = 5) (ANOVA followed by *t*-Student–Neuman–Keuls posthoc test); ^a^ *p* < 0.05, significantly different from the negative control (NC) group (vehicle); ^b^ *p* < 0.05, significantly different from the diazepam (DZP) group; ^c^ *p* < 0.05, significantly different from the quercetin (QUR) group; ^d^ *p* < 0.05, significantly different from the ascorbic acid (AA) group; FLU: Flumazenil.

**Table 2:** The best three results of a molecular docking study of quercetin (QUR) with GABA receptors.

Protein (Receptor)	Binding Affinity (Kcal/mol)	No of H-Bond	H-Bond Residues	Bond Length (Å)	Other Bond Residues
GABA (A5)	−6.9	1	Glu327	2.94	Ile391, Trp320
GABA (B1)	−8.4	3	Arg571 Glu745 Ser813	2.88 2.39 1.98	Ala819, Leu633
GABA (B2)	−8.2	4	Arg556 Gln720 Glu646 Ser710	2.14 2.90 2.19 2.12	His647, Phe537, Pro717

**Table 3:** The best three results of a molecular docking study of ascorbic acid (AA) with GABA receptors.

Protein (Receptor)	Binding Affinity (Kcal/mol)	No. of H-Bond	H-Bond Residues	Bond Length (Å)	Other Bond Residues
GABA (A2)	−5.0	4	Ile37 Glu39 Ser36 Thr38	2.41 2.12 1.89 2.37	
GABA (B1)	−5.5	3	Arg652 Glu745 Leu573	2.70 2.43 2.49	Leu635
GABA (B2)	−5.2	4	Arg556 Gln720 His647 Leu539	2.84 2.51 2.75 2.44	

**Table 4:** The calculated binding energies of AA and QUR against targets (GABA (A2), GABA (A5), GABA (B1), and GABA (B2)) complexes.

Complex Name	∆G_vdW_ (kJ/mol)	∆G_elec_ (kJ/mol)	∆G_pol_ (kJ/mol)	∆G_nonpol_ (kJ/mol)	∆E (MM-PBSA) (kJ/mol)
GABA A2-AA	−65.844	−48.201	97.412	−9.442	−26.074
GABA A2-QUR	−68.791	−7.920	53.211	−10.038	−33.538
GABA A5-AA	−48.923	−45.689	70.275	−7.732	−32.068
GABA A5-QUR	−61.891	−38.976	82.465	−8.669	−27.071
GABA B1-AA	−61.664	−31.617	78.994	−7.740	−22.027
GABA B1-QUR	−76.362	−78.516	142.703	−12.908	−25.083
GABA B2-AA	−75.571	−46.703	122.131	−10.228	−14.629
GABA B2-QUR	−82.083	−52.631	128.317	−10.453	−16.850

**Table 5:** Groups pretreated at 10 mL/kg of intraperitoneally (i.p.) administered phenobarbital sodium (30 mg/kg, i.p.) in Swiss mice (*n* = 5).

Treatment Groups	Description	Dose
Gr.-I: NC	Distilled water	10 mL/kg
Gr.-II: DZP	Diazepam (Standard 1: Benzodiazepine receptor agonist)	2 mg/kg
Gr.-III: QUR	Quercetin (Test sample 1)	50 mg/kg
Gr.-IV: AA	Ascorbic acid (Test sample 2)	25 mg/kg
Gr.-V: DZP + QUR	Diazepam + Quercetin	2 mg/kg + 50 mg/kg
Gr.-VI: DZP + AA	Diazepam + Ascorbic acid	2 mg/kg + 25 mg/kg
Gr.-VII: DZP + AA + QUR	Diazepam + Ascorbic acid + Quercetin	2 mg/kg + 25 mg/kg + 50 mg/kg
Gr.-VIII: FLU	Flumazenil (Standard 2: Benzodiazepine receptor antagonist)	2 mg/kg
Gr.-X: QUR + FLU	Quercetin + Flumazenil	50 mg/kg + 2 mg/kg
Gr.-IX: AA + FLU	Ascorbic acid + Flumazenil	25 mg/kg + 2 mg/kg

## Data Availability

Not applicable.
